# Seasonal Variation in Pasture Availability During the Fattening Period Influences Lipid Profile and Nutritional Quality in the Semi-Extensive Production of Iberian Pigs

**DOI:** 10.3390/ani16101531

**Published:** 2026-05-16

**Authors:** José M. Martínez-Torres, Juan Florencio Tejeda, Juan M. García Casco, Elena González

**Affiliations:** 1 Food Science and Technology, Escuela de Ingenierías Agrarias, Universidad de Extremadura, Avda. Adolfo Suárez s/n, 06007 Badajoz, Spain; jmtorres@unex.es; 2 Research University Institute of Agricultural Resources (INURA), Avda. de Elvas s/n, Campus Universitario, 06006 Badajoz, Spain; malena@unex.es; 3 Iberian Pig R&D Center, Instituto Nacional de Investigación y Tecnología Agraria y Alimentaria-Consejo Superior de Investigaciones Científicas (INIA-CSIC), Carretera EX-101, Km 4.7, 06300 Zafra, Spain; garcia.juan@inia.csic.es; 4 Animal Production, Escuela de Ingenierías Agrarias, Universidad de Extremadura, Avda. Adolfo Suárez, 06007 Badajoz, Spain

**Keywords:** Iberian pig, semi-extensive production, seasonal fattening, fatty acids, meat quality, adipose tissue, intramuscular fat, neophytadiene, grazing

## Abstract

Iberian pigs reared in a semi-extensive outdoor system, known as *cebo de campo*, have their commercial feed supplemented with natural pasture. However, the availability and quality of pastures vary seasonally under the Mediterranean conditions of the southwestern Iberian Peninsula. This study evaluates the effect of the seasonal fattening period on fatty acid content and nutritional quality indices (atherogenic index, thrombogenic index, hypocholesterolemic/hypercholesterolemic ratio, and healthy fatty index) in subcutaneous adipose tissue and intramuscular fat from 258 purebred Iberian pigs raised under the *cebo de campo* system during three periods: winter–spring (December–April, *n* = 63), spring–summer (May–August, *n* = 99), and summer–autumn (August–November, *n* = 96). Winter–spring pigs exhibited significantly higher monounsaturated and polyunsaturated fatty acids and lower saturated fatty acids compared to spring–summer and summer–autumn groups in subcutaneous adipose tissue. Nutritional quality indices were more favorable in winter–spring pigs, indicating superior cardiovascular health benefits. Principal component analysis revealed clear seasonal group separation, with the first two components explaining 88.51% of the variance in subcutaneous fat. Significantly higher neophytadiene concentrations, a biomarker of pasture intake, in winter–spring pigs confirmed greater pasture consumption. These findings demonstrate that seasonal factors significantly influence the nutritional quality of meat produced in *cebo de campo* Iberian pig systems. This influence is much clearer in subcutaneous adipose tissue than intramuscular fat, as winter–spring fattening is associated with a more favorable lipid profile.

## Simple Summary

Iberian pigs raised under a semi-extensive outdoor system complement a commercial feed-based diet with natural pasture available in their grazing area. Because production is located in the southwestern Iberian Peninsula under a Mediterranean climate, pasture availability and quality can fluctuate seasonally due to prevailing weather conditions. This study investigates whether the fattening season influences the nutritional quality of adipose tissue and muscle in these pigs. We analyzed samples from 258 Iberian pigs fattened in three different seasons: winter–spring, spring–summer, and summer–autumn. Pigs fattened in the winter–spring period showed higher contents of unsaturated and lower contents of saturated fatty acids than pigs fattened in spring–summer or summer–autumn periods, together with improved health-related indices (atherogenic, thrombogenic and healthy fatty indices, and hypocholesterolemic/hypercholesterolemic ratio). This is more aligned with cardiovascular health-oriented dietary recommendations; the clearest differences were observed in subcutaneous adipose tissue. Higher concentrations of neophytadiene, a biomarker indicative of pasture intake that accumulates in pig tissues, are associated with consumption of fresh grass during the winter–spring period. These findings indicate that the season in which pigs are raised has significant impact on meat quality and health benefits for consumers. This information could help farmers optimize production timing for semi-extensive outdoor systems.

## 1. Introduction

Nutritional science has evolved from emphasizing total fat reduction to assessing fat quality, recognizing that the type of fatty acid and proportion determine health implications rather than absolute fat content [1,2]. Several studies focus on the balance between saturated (SFA), monounsaturated (MUFA), and polyunsaturated (PUFA) fatty acids in determining cardiovascular health outcomes [3,4,5].

In pork, lipids are distributed, among others, across subcutaneous adipose tissue (SAT) and intramuscular fat (IMF), which differ substantially in fatty acid composition [6]. For traditional products from pig breeds based on less highly selected “heritage breeds” (e.g., Iberian pig), such as dry-cured Iberian ham, consumers typically ingest both tissues together, making the integrated fatty acid profile nutritionally significant and a reflection of actual consumption patterns [7].

Production, processing, and marketing of meat and dry-cured products from Iberian pigs are regulated by a National Quality Standard (NQS) enacted by the Spanish government [8]. Meat products derived from Iberian pigs, particularly dry-cured hams and loins, are greatly valued by Spanish consumers due to their distinctive sensory characteristics [9]. Genetics and diet are the main factors that affect the quality of Iberian pig meat and meat products [10,11]. Regarding diet, the NQS Spanish legislation establishes three main categories for Iberian products, known as *bellota* (Iberian pigs exclusively fed with natural pasture and acorns under extensive conditions), *cebo de campo* (pigs that receive mixed feeding from concentrate feeds complemented with natural pasture and acorns, if available, under semi-extensive outdoor rearing conditions), and *cebo* (pigs exclusively fed with concentrate feeds in confinement). Production of pigs classified as *cebo de campo* has increased in recent years, accounting for approximately 15% of the total Iberian pig production in the Iberian Peninsula [12]. Two main factors explain the increasing trend of producing this type of animals: the positive impact associated with rearing animals under extensive or semi-extensive conditions, and the possibility of finishing animals throughout the year.

Beyond the well-established importance of assessing Iberian pig production conditions in relation to the sensory quality of meat products [13], there has been growing interest in recent years in evaluating the effects of these factors on the nutritional quality of both Iberian pig meat and meat products [14,15]. Fatty acid composition in meat responds dynamically to environmental and management factors. This fact is particularly important in the case of *cebo de campo* pigs, which are raised outdoors with access to natural forage and supplementary concentrated feed. Seasonal variations in temperature, forage availability, and feed composition during different seasons can substantially influence lipid deposition and fatty acid composition [16,17]. In relation to fatty acid composition, the assessment of nutritional lipid indices has gained considerable attention in recent years due to their relevance in determining the health-promoting properties of meat lipids [18,19]. Accordingly, several indices have been proposed in the literature, including the atherogenic index (AI) and thrombogenic index (TI) [20], the hypocholesterolemic/hypercholesterolemic (h/H) ratio [21], the healthy fatty index (HFI) [22], and the n-6/n-3 ratio [14]. In addition, previous studies have provided evidence on the heterogeneity of Iberian pig production under extensive conditions, with variations between different production seasons and geographical areas, and intensive conditions [16,23]. These production differences lead to significant variations in the accumulation of various unsaponifiable lipid compounds in muscle and subcutaneous fat. Compounds such as tocopherols and branched hydrocarbons are associated with the intake of grass and acorns in free-range conditions. Thus, they can be considered potential quality indicators in Iberian pig meat and meat products [24,25,26]. Neophytadiene is a branched hydrocarbon that accumulates specifically in tissues of animals consuming fresh grass, suggesting its suitability as a qualitative biomarker of pasture intake in Iberian pig production systems [24,27]. However, to the best of our knowledge, a comprehensive evaluation of the seasonal effects of the fattening period on *cebo de campo* Iberian pigs has not been fully investigated, particularly for subcutaneous and intramuscular lipid indices and feeding-related biomarkers.

Thus, we hypothesize that seasonal variations in pasture availability significantly influence lipid deposition patterns in *cebo de campo* Iberian pigs. Therefore, the objective of this study was to characterize the fatty acid contents and nutritional quality indices (AI, TI, h/H ratio, n-6/n-3 ratio, and HFI) of Iberian pork across different fattening seasons. This was conducted through an integrated analysis of intramuscular fat and subcutaneous adipose tissue, in order to provide evidence-based nutritional information for traditional Iberian pork products.

## 2. Materials and Methods

### 2.1. Animals and Experimental Design

**Figure 1:**
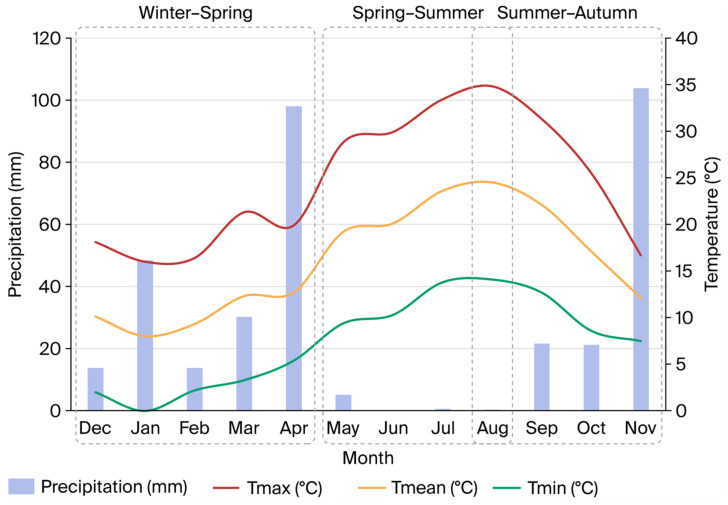
Monthly precipitation (mm) and maximum and minimum air temperatures (°C) during the study period, recorded at the Spanish Meteorological Agency (AEMET) weather station in Aroche (Huelva, Spain; station ID 4527X). Source: AEMET [28].

A total of 258 castrated male purebred Iberian pigs of the *Retinto* line were used in this study. Animals were fattened in the southwestern Iberian Peninsula at the agroforestry estate *La Contienda,* in Aroche, Huelva, Spain (approximately 38.05542° N latitude and −6.93554° W longitude), under the semi-extensive system known as *cebo de campo* and in agreement with the Iberian pig NQS [8]. During the fattening period, each group of pigs was kept in a separate free-range paddock (~10 ha per group) within the same Mediterranean *dehesa* (an herbaceous pasture with scarce holm oaks), ensuring comparable pasture conditions across groups. All animals had continuous access to the outdoors and shelter availability and were exposed to ambient climatic conditions throughout the trial. Three independent experimental groups were established based on the fattening season for these animals: winter–spring (*n* = 63), where Iberian pigs were fattened from December to April; spring–summer (*n* = 99), where pigs were fattened from May to August; and summer–autumn (*n* = 96), where pigs were fattened from August to November. To characterize seasonal environmental conditions, monthly temperature and precipitation data were obtained from the *Agencia Estatal de Meteorología* (AEMET; Spanish Meteorological Agency) [28] weather station in Aroche (Huelva, Spain; station ID 4527X), which is the nearest official station to the farm. The area exhibits a Mediterranean climate, with rainfall mainly concentrated in autumn–winter and a marked summer drought, together with the highest maximum temperatures in mid–late summer. As shown in Figure 1, precipitation is higher during late autumn–winter and decreases sharply in summer, when maximum temperatures are reached. Overall, these conditions support marked seasonal differences in pasture growth and availability for pig grazing across the three fattening periods studied (winter–spring, spring–summer and summer–autumn).

Pigs were randomly allocated into the three study groups, starting the fattening period at an average body weight of 97 kg (with an average age of 9.5 months). They were slaughtered at approximately 165 kg and 12.5 months of age (after approximately 125 days on trial). The three groups of pigs were offered the same commercial concentrate (11.68% crude protein; 3.5% crude fat; 51.6% starch; main fatty acids: 36.5% linoleic, 33.2% oleic, 18.9% palmitic), formulated to provide 17.15 MJ metabolizable energy/kg dry matter, according to the *Fundación Española para el Desarrollo de la Nutrición Animal (FEDNA)* [29]. Feed and water were provided *ad libitum*. Moreover, the pigs had access to pasture, which varied in availability across seasons. As pigs were fed in semi-extensive conditions, it was not possible to measure their pasture intake directly. Seasonal differences in pasture availability and nutritional quality were therefore interpreted in the context of the characteristic seasonal dynamics of Mediterranean pastures under comparable climatic conditions. Two slaughter time points were established for each experimental group. Pigs from the winter–spring group were slaughtered at the end of March (*n* = 48) and in mid-April (*n* = 15). Animals from the spring–summer group were slaughtered at the end of August (*n* = 80) and in early September (*n* = 19). Finally, pigs from the summer–autumn group were slaughtered at the end of October (*n* = 78) and in mid-November (*n* = 18). Immediately after slaughter, subcutaneous adipose tissue and muscle (*longissimus lumborum*) samples of each pig were collected from the right half-carcass, vacuum-packed, and stored at −20 °C until analysis.

### 2.2. Lipid Extraction and Fatty Acid Analysis

Subcutaneous adipose tissue samples (3 cm × 3 cm), containing skin, fat, and a small portion of lean meat, were obtained 10 cm above the tail insertion area of the carcasses following the procedure established by the Spanish Ministry of Agriculture, Fisheries, and Food (Order PRE/3844/2004) [30]. Additionally, samples from the *longissimus lumborum* muscle (approximately 300 g) were collected from each pig. All the samples were individually placed in plastic bags, vacuum-sealed, and stored at −20 °C until chemical analysis. Prior to analysis of fatty acids, both subcutaneous and muscle samples were ground using a commercial grinder (Waldmann, Paris, France).

Lipids from SAT samples were extracted using a microwave according to the method described by De Pedro et al. [31]. IMF was extracted and quantified according to the method of Bligh and Dyer [32]. For both SAT and IMF, fatty acid methyl esters (FAMEs) were prepared from the extracted lipids via acidic transesterification, following the method of Sandler and Karo [33], using sodium metal (0.1 N) and sulfuric acid (5% sulfuric acid in methanol). The resulting FAMEs were analyzed using gas chromatography with a Hewlett–Packard HP-4890 Series II GC equipped with a split/splitless injector and a flame-ionization detector (Hewlett-Packard, Palo Alto, CA, USA). The derivatives were separated using a Carbowax 20 fused-silica capillary column (30 m length, 0.25 mm internal diameter, 0.25 μm film thickness; Agilent, Santa Clara, CA, USA). For the analysis, the oven temperature was maintained at 200 °C for 25 min. Injector and detector temperatures were maintained at 250 °C. The split mode (ratio 1:50) was used at a flow rate of 1.8 mL/min, using nitrogen as the carrier gas. Individual fatty acids were identified by comparing retention times with those of standard reference mixtures (Sigma Chemical Co., St. Louis, MO, USA). Calibration curves for all 16 fatty acids identified were previously established in our laboratory using external fatty acid standards at different concentrations, and quantification was based on the relationship between peak area and concentration. Results were expressed as mg/g of tissues, using tridecanoic acid (C13:0) as the internal standard.

### 2.3. Nutritional Indices

Table 1 presents the fatty acid-related indices used in this study, alongside detailed formulas, which, according to the literature, may have implications for the nutritional quality of Iberian pig meat. In addition to classical indices, such as the total sums of saturated, monounsaturated, polyunsaturated and unsaturated fatty acids (SFAs, MUFAs, PUFAs and UFAs, respectively), as well as total n-6 and n-3 polyunsaturated fatty acids, the ratios between these groups (n-6/n-3, PUFA/SFA, MUFA/SFA, and UFA/SFA) were evaluated. Moreover, other nutritional indices were examined, such as the AI, TI, the ratio of h/H, and the HFI, which are widely used to evaluate the nutritional quality of lipid in meat and meat products. In pork, these indices provide an integrative assessment of the fatty acid profile, reflecting the balance between fatty acids considered to have potentially beneficial or adverse effects on lipid metabolism and cardiovascular risk. Their use results in a more functional interpretation of the fatty acid composition beyond individual fatty acids. However, it should be noted that these indices are indirect indicators and do not represent direct measures of human health outcomes, but rather proxies based on known relationships between fatty acid composition and cardiovascular risk.

The AI and TI were calculated according to Ulbricht and Southgate [20]. The AI indicates the relationship between the sums of the main saturated fatty acid (C12:0, C14:0 and C16:0, with the exception of C18:0) and the sum of total unsaturated fatty acids; by contrast, the TI was calculated as the ratio between the pro-thrombogenic fatty acids (C14:0, C16:0, and C18:0) and the anti-thrombogenic fatty acids (MUFA and the n-3 and n-6 families).

The h/H index characterizes the relationships between hypocholesterolemic fatty acids (C18:1 and PUFA) and hypercholesterolemic fatty acids (C12:0, C14:0, and C16:0), according to Mierliţă [21]. Finally, the HFI was calculated according to the formula proposed by Dal Bosco et al. [22].

### 2.4. Neophytadiene Analysis

The hydrocarbon fraction of SAT was analyzed according to the procedure described by Tejeda et al. [24]. Briefly, 2 g of each sample was subjected to saponification by refluxing for 15 min in 70 mL of ethanolic potassium hydroxide (15%, *w*/*v*). The unsaponifiable fraction was subsequently recovered by extraction with 70 mL of hexane. Total hydrocarbons were purified using a 500 mg aminopropyl mini column eluted with 30 mL of hexane. Following solvent evaporation to dryness under reduced pressure, the resulting residue was reconstituted in 1 mL of hexane and prepared for gas chromatographic analysis.

Neophytadiene identification and quantification were carried out using an Agilent 6890 gas chromatograph coupled to an Agilent 5973 mass-selective detector (Agilent Technologies, Santa Clara, CA, USA) and fitted with an HP-1 capillary column (12 m × 0.2 mm internal diameter × 0.33 μm film thickness) containing a methyl siloxane stationary phase (Agilent Technologies, Santa Clara, CA, USA). The oven temperature program started at 100 °C and increased to 196 °C at a rate of 6 °C/min, followed by a rise from 196 to 280 °C at 30 °C/min, with a final hold of 15 min at 280 °C. The injector temperature was maintained at 260 °C, while the GC–MS transfer line was set at 280 °C. Analyses were performed in split mode with a split ratio of 1:25, an inlet pressure of 14 psi, and an injection volume of 2 μL.

The mass spectrometer operated under electron impact ionization at 70 eV, with a multiplier voltage of 1756 V. Data acquisition was performed at a rate of 1 scan/s over a mass-to-charge (*m*/*z*) range of 40–300. Compound identification was achieved by comparing the obtained mass spectra with those of authentic standards and spectra available in the National Institute of Standards and Technology library. Quantification was performed using n-nonadecane (n-C_19_) as an internal standard, and results were expressed as relative area units, calculated as peak area × 100 divided by the internal standard peak area.

### 2.5. Statistical Analysis

An individual pig was considered the experimental unit. Each sample of SAT and IMF from each animal was analyzed in technical duplicate, which was not considered an independent experimental observation. Data were expressed as the mean ± standard error. The statistical model included fattening season as the fixed effect. Because the animals were homogeneous in terms of sex and genetic line, they were managed under the same production system, and were offered the same commercial concentrate. One-way analysis of variance (ANOVA) was used to evaluate the effect of season on each response variable. Within each seasonal group, animals were slaughtered at two time points; however, including slaughter date or body weight in the model did not improve model fit. Data were analyzed using the General Linear Model (GLM) procedure of SPSS (v.22; SPSS Inc., Chicago, IL, USA). Prior to analysis, ANOVA assumptions were checked by evaluating data distribution, residual behavior, and homogeneity of variances. When the main effect was significant (*p* < 0.05), means were compared using Tukey’s post hoc test. Principal component analysis (PCA) was performed on both SAT and IMF to explore general patterns in lipid composition and nutritional quality, including the main fatty acid composition variables and health-related lipid indices (AI, TI, h/H, n-6/n-3, and HFI).

### 2.6. Ethics Statement

This experiment was conducted under standard commercial pig production conditions, and animals were subjected only to routine husbandry and feeding practices inherent to the production system. No additional procedures requiring authorization under Directive 2010/63/EU and Royal Decree 53/2013 were performed; therefore, specific ethical approval was not required. Animal management complied with Council Directive 2008/120/EC and the corresponding Spanish legislation on pig welfare and production conditions.

## 3. Results

### 3.1. Fatty Acid Content

Table 2 shows the fatty acid profile of subcutaneous adipose tissue and *longissimus lumborum* muscle from Iberian pigs fed under the *cebo de campo* feeding system. The results provide evidence that the fatty acid composition in both tissues, SAT and IMF, was significantly influenced by the seasonality of the fattening period, with a noticeable overall difference between the winter–spring group and those corresponding to spring–summer and summer–autumn groups.

Regarding SAT composition, palmitic acid (C16:0) was the most abundant among the SFAs (197.58–214.45 mg/g), followed by stearic acid (C18:0) (98.87–114.14 mg/g), and myristic acid (C14:0) (11.25–12.66 mg/g), all of which had significant lower contents (*p* < 0.001) in animals fed in winter–spring period compared to pigs fed both in spring–summer and summer–autumn. The latter two groups showed similar values.

Considering MUFAs, oleic acid (C18:1 n-9) was the predominant fatty acid in SAT across all seasons, accounting for around 50% of all fatty acids identified in this tissue. Pigs fattened in the winter–spring period exhibited a significantly higher oleic acid content (453.07 mg/g) compared to animals in spring–summer (430.99 mg/g) and summer–autumn (427.40 mg/g) (*p* < 0.001). Eicosenoic acid (C20:1 n-9) (12.67–14.13 mg/g) and heptadecenoic acid (C17:1) (3.58–4.02 mg/g) followed a similar trend, with winter–spring animals showing significantly higher concentrations (*p* < 0.001) than the other two groups. However, concentrations of palmitoleic acid (C16:1) (21.95–22.67 mg/g) remained stable across seasons, with no significant differences detected (*p* > 0.05). Finally, linoleic acid (C18:2 n-6) was the most commonly observed PUFA, with winter–spring pigs showing significantly higher values (71.42 mg/g) compared to animals fattened in the spring–summer (67.33 mg/g) and summer–autumn (67.67 mg/g) (*p* < 0.001). A similar pattern was observed in the other PUFA identified, except for arachidonic acid (C20:4 n-6), which showed significantly higher concentrations in the summer–autumn (1.28 mg/g) compared to winter–spring (1.20 mg/g) and spring–summer periods (1.18 mg/g) (*p* < 0.001).

The fatty acid profile of the IMF tissue also showed significant variations according to the fattening season, and was characterized by a predominance of MUFAs, followed by SFAs and PUFAs. As previously described in SAT, C16:0 was the most abundant SFA in muscle (11.01–16.05 mg/g), followed by C18:0 (4.70–6.79 mg/g), while other minor SFAs (C14:0, C17:0, and C20:0) accounted for less than 1 mg/g. In all cases, winter–spring pigs exhibited significantly higher (*p* < 0.001) contents than the other two groups. Oleic acid (C18:1 n-9) was the predominant fatty acid in the intramuscular fat across all seasons; by contrast, winter–spring pigs presented significantly higher content (30.51 mg/g) compared to spring–summer (20.93 mg/g) and summer–autumn (21.72 mg/g) animals (*p* < 0.001). C16:1, C17:1, and C20:1 n-9 fatty acids showed a similar trend, as previously described in oleic acid. Among the PUFAs, C18:2 n-6, C18:3 n-3, and C20:2 n-9 also showed significantly higher levels in winter–spring compared to the other two groups. In contrast, C20:3 n-6, C20:4 n-6, and C20:3 n-3 showed no significant seasonal variations, with concentrations ranging from 0.05 to 0.40 mg/g across all groups.

### 3.2. Nutritional Lipid Quality Indices

The effect of the fattening season on the nutritional lipid quality indices of SAT and IMF in *cebo de campo* Iberian pigs is presented in Table 3. As previously described in fatty acid content, the nutritional indices evaluated showed significant differences between the three seasons studied, in both adipose and muscle tissues analyzed. However, in this case, the differences were more noticeable in the SAT than in the *longissimus lumborum* muscle.

In adipose tissue all the evaluated indices differed significantly (*p* < 0.001) among the three experimental treatments, except for the total fat content (which ranged from 892.47 to 893.69 mg/g). The winter–spring production group consistently differed from the other two groups. Significant seasonal effects were observed for all fatty acid groups. The total SFA content was significantly lower in animals fattened in the winter–spring compared to spring–summer and summer–autumn (346.71 mg/g). No differences were found between the latter two seasons. Conversely, MUFAs, PUFAs, UFAs, n-6, and n-3 exhibited an opposite trend. All fatty acid ratios studied (PUFA/SFA, MUFA/SFA, and UFA/SFA) were significantly affected by season (*p* < 0.001), with significantly higher values observed in winter–spring than in spring–summer and summer–autumn periods, whereas the n-6/n-3 ratio showed the opposite trend. Nutritional quality indices calculated in SAT revealed highly significant seasonal effects (*p* < 0.001). The AI and TI indices were significantly lower in winter–spring (0.42 and 1.01, respectively) compared to spring–summer (0.48 and 1.18) and summer–autumn (0.48 and 1.20). However, the h/H ratio and the HFI were significantly higher in winter–spring (2.59 and 2.27, respectively) compared to spring–summer (2.28 and 2.07) and summer–autumn (2.24 and 2.06). For all four nutritional quality indices, no significant differences were observed between fattening periods in the spring–summer and summer–autumn periods.

Regarding the effect of seasonality on the nutritional lipid quality indices of IMF, the total intramuscular fat content showed significant seasonal variation (*p* < 0.001), with winter–spring pigs exhibiting substantially higher values (60.93 mg/g) compared to spring–summer (42.60 mg/g) and summer–autumn (44.88 mg/g) groups. The fatty acid group composition (SFAs, MUFAs, PUFAs, UFAs, total n-6, and n-3), and the HFI followed a similar pattern, with significantly higher contents in winter–spring group than in the other two groups. By contrast, the n-6/n-3 and PUFA/SFA ratios showed the opposite trend. However, the MUFA/SFA and UFA/SFA ratios, as well as the AI, TI and h/H indices, did not show significant seasonal variation (*p* > 0.05). Finally, for the calculated nutritional quality indices in muscle, the results revealed that only HFI was affected by the season in which pigs were fattened (*p* < 0.001), with winter–spring pigs exhibiting the highest values (1.40) compared to spring–summer (1.24) and summer–autumn (1.27) groups.

### 3.3. Principal Component Analysis

**Figure 2:**
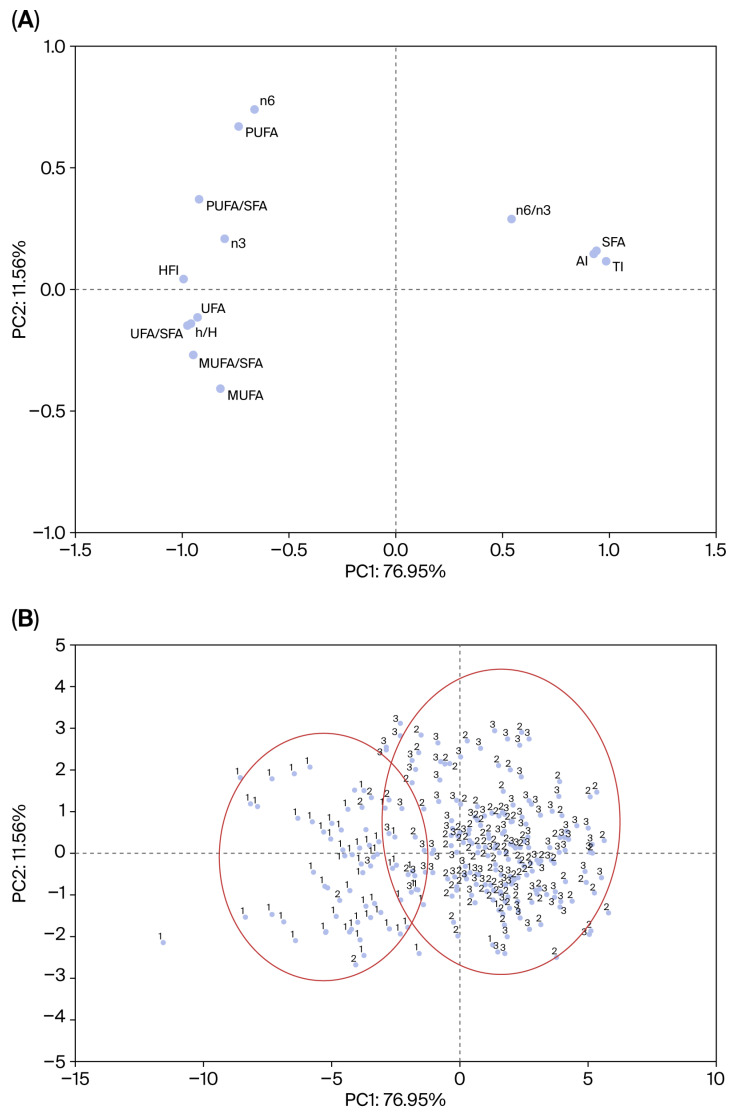
Principal component analysis of lipid composition and nutritional quality indices in subcutaneous adipose tissue from Iberian pigs reared under the *cebo de campo* system during different fattening seasons. (**A**) Loading plot showing variable contributions to PC1 and PC2. (**B**) Score plot of individual pigs by season: 1 = winter–spring (n = 63); 2 = spring–summer (n = 99); 3 = summer–autumn (n = 96). Ellipses show 95% confidence intervals. Clear separation is observed along PC1.

The PCA results for SAT are presented in the main text (Figure 2), as this tissue showed the clearest multivariate discrimination among seasons. This is in agreement with the univariate analyses, which revealed more marked seasonal differences in SAT than in IMF.

The first two principal components (PC1 and PC2) of the SAT explained 88.5% of the total variance (76.95% and 11.56%, respectively). The loading plot (Figure 2A) revealed that PC1 was primarily associated with the degree of unsaturated fatty acid. Variables representing a healthier lipid profile (PUFAs, MUFAs, HFI, h/H and UFA/SFA ratio) loaded negatively on PC1, while variables associated with cardiovascular risk (SFAs, AI, TI, and n-6/n-3 ratio) loaded positively. PC2 was mainly related to the balance between PUFAs (positive loadings) and MUFAs (negative loadings). The score plot (Figure 2B) showed clear separation among the three seasonal groups along PC1. The winter–spring group clustered on the negative side of PC1, indicating a lipid profile characterized by a higher unsaturated content of fatty acids and more favorable nutritional indices. In contrast, the spring–summer and summer–autumn groups were positioned on the positive side of PC1, with the summer–autumn group showing the highest SFA content and least favorable AI and TI values.

In the IMF, the first two principal components explained 82.16% of the total variance (49.04% and 33.12%, respectively). However, despite this relatively high cumulative variance, the score distribution showed substantial overlap among the seasonal groups, indicating that the overall IMF lipid profile provided weaker discrimination according to the fattening season.

### 3.4. Neophytadiene Contents

**Figure 3:**
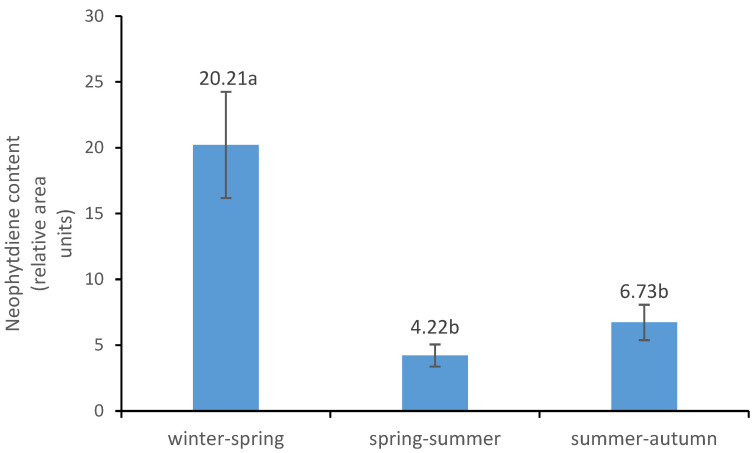
Effect of seasonal fattening period on the neophytadiene content (relative area units) of subcutaneous adipose tissue (SAT). For each seasonal group, the mean and the standard error bar is presented. Different letters between mean values indicate significant differences among batches at *p* < 0.001.

Neophytadiene concentrations (expressed as relative area units) in SAT are shown in Figure 3. Iberian pigs fattened during winter–spring (20.21) exhibited a significantly higher neophytadiene content than pigs fattened during spring–summer (4.22) and summer–autumn (6.73) periods (*p* < 0.001). No significant differences (*p* > 0.05) were found between these last two groups of animals.

## 4. Discussion

Seasonal fattening under the semi-extensive *cebo de campo* system was associated with differences in lipid composition and nutritional quality traits in Iberian pigs, with much clearer results in SAT than in IMF.

The general fatty acid pattern observed in both tissues was consistent with previous reports in *cebo de campo* Iberian pigs [34,35], in which oleic acid was the predominant fatty acid, followed by palmitic and stearic acids; by contrast, linoleic acid is the main PUFA [16]. As widely reported, the fatty acid composition of pig tissues is mainly determined by diet [36]; however, other factors such as genetics, sex, age, slaughter weight, physical activity, and environmental conditions may also modulate the final lipid profile [37,38,39,40,41].

Under NQS Spanish legislation [8], the *cebo de campo* fattening system can be carried out throughout the year, which means that pigs may be exposed to markedly different feeding and environmental conditions depending on the season. Considering the large climatic variations between summer and winter seasons in the southwestern Iberian Peninsula, this could result in different fat composition in Iberian pig [42,43]. The present results are consistent with this view, since pigs fattened during winter–spring showed differences in the fatty acid content of both SAT and IMF for many of the fatty acids analyzed. Differences were observed in pigs fattened during spring–summer and summer–autumn periods. These seasonal differences could be explained, at least in part, by differences in pasture availability. Under Mediterranean conditions, the season of the year strongly affects pasture quantity and quality in outdoor free-range areas where *cebo de campo* pigs are raised, with herbage production generally peaking in spring and declining during the summer dry period [44]. In addition to the commercial concentrate supplied during fattening, fresh pasture constitutes a relevant complementary feed resource in this semi-extensive production system [45] and a source of α-linolenic acid (C18:3 n-3), one of the predominant fatty acids in herbage [46]. In monogastric species such as pigs, dietary fatty acids can be incorporated relatively directly into tissue lipids [26]. Accordingly, the higher C18:3 n-3 concentrations observed in winter–spring pigs are consistent with greater access to fresh pasture during that period. Moreover, seasonal differences in both pasture availability and pasture quality may have contributed to the tissue differences observed in the present study [47]. Rey et al. [26] and Lebret et al. [43] suggested that other factors related to the rearing system, beyond grass intake, are likely involved in the differences in fatty acid composition of Iberian pig tissues. Environmental conditions, particularly the marked temperature differences between seasons, may also influence fat composition. In this regard, Bee et al. [48] reported that commercial pigs reared outdoors during the winter (5 °C) showed significantly lower proportions of saturated fatty acids than pigs reared indoors at 22 °C. Similarly, Kouba et al. [49] also provided evidence that environmental temperatures can affect the SFA and MUFA composition of pigs, possibly through changes in stearoyl-CoA desaturase activity, which decreases as temperature increased. Stearoyl-CoA desaturase, a key lipogenic enzyme, catalyzes the conversion of saturated fatty acids into monounsaturated fatty acids. Thus, changes in this enzyme activity may affect the balance between SFA and MUFA in pig tissues. Therefore, both seasonal conditions and pasture availability may be plausible causes of the seasonal differences observed in *cebo de campo* pigs in the present study, with potential implications not only for the nutritional quality of fat and meat products, but also for their technological and sensory quality [26,50].

A particularly relevant aspect of the present results is that the seasonal response was much clearer in SAT than in IMF. This pattern was reflected not only in the number and magnitude of significant differences among batches, but also from the multivariate analysis, which showed a clear separation of seasonal groups in adipose tissue. This tissue-dependent response is biologically plausible and consistent with previous studies evaluating tissue-specific regulations of lipid metabolism in pigs [51]. Subcutaneous fat exhibits a higher capacity for lipid deposition and turnover, as well as greater responsiveness to dietary changes, largely driven by increased activity of lipogenic enzymes and lipoprotein lipase. This facilitates the uptake of circulating fatty acids [52]. In contrast, intramuscular fat is more metabolically constrained, with a lower rate of lipid turnover and a higher proportion of structural lipids, such as phospholipids, which are less influenced by short-term dietary variations [18]. Furthermore, adipocytes in subcutaneous fat tend to show greater hypertrophy and hyperplasia in response to nutritional conditions, whereas intramuscular adipocytes are smaller and more stable [53]. In line with these tissue-specific differences, fatty acid deposition in pigs is strongly depot-dependent, and subcutaneous adipose tissue generally exhibits a higher unsaturated fatty acid profile than intramuscular fat and other more internal depots [18]. From a human nutrition perspective, expressing fatty acids as mg/g tissue is informative because it reflects the absolute amount of each fatty acid present in the edible product. However, in the IMF, these values should be interpreted with caution, since the higher total intramuscular fat content observed in winter–spring pigs may have contributed, at least in part, to the higher absolute contents of several fatty acids detected in muscle. Therefore, in the IMF, differences expressed on a tissue basis do not necessarily imply proportional changes of the same magnitude in the fatty acid composition of the lipid fraction. Overall, these results are consistent with the fact that SAT is a more sensitive tissue than IMF for detecting differences associated with the seasonal fattening conditions of *cebo de campo* pigs.

The nutritional quality indices should be interpreted considering the marked differences between SAT and IMF. Since the total content of UFA is considered a representative indicator of lipid quality in pig tissues [18], the higher proportions of MUFAs and PUFAs found in subcutaneous adipose tissue could explain the more favorable values of AI, TI, h/H, and HFI observed there compared with muscle. This is in agreement with previous studies [54]. In this context, as suggested by Chen and Liu [55], the interpretation of nutritional indices should also consider the total fat content of the tissue evaluated, since tissue-specific differences in fat deposition may influence their biological meaning. Within this tissue-dependent framework, differences associated with the fattening season were more pronounced in SAT than in IMF. In SAT, winter–spring pigs showed lower SFA proportions and more favorable values of AI, TI, h/H, and HFI than spring–summer and summer–autumn pigs, indicating a more desirable lipid profile from a cardiovascular health perspective [55]. These findings are consistent with previous reports describing seasonal variations in the fatty acid composition of pig tissues [16] and, consequently, in atherogenic indices. This highlights the importance of considering seasonality as a factor associated with the nutritional quality of pig fat, which may be related to seasonal variation in diet quality and feed resources [56].

The PCA provided an integrative view of these relationships and reinforced the interpretation derived from the univariate analyses. In SAT, the first principal component was mainly associated with the degree of fatty acid unsaturation and the contrast between favorable lipid quality variables (PUFAs, MUFAs, h/H, HFI, and UFA/SFA ratio) and less desirable ones (SFAs, AI, TI, and n-6/n-3 ratio). From a biological perspective, this axis may reflect a gradient from a more desaturated adipose lipid profile to a relatively more saturated one under different seasonal conditions, likely associated with differences in the incorporation of dietary unsaturated fatty acids and in desaturation-related lipid metabolism [57]. In turn, PC2 seemed to capture a secondary variation within the unsaturated fraction, mainly related to the relative contribution of PUFAs and MUFAs. The clear clustering of winter–spring pigs on the side of the plot associated with higher unsaturation and more favorable nutritional indices could indicate that seasonal effects are not just restricted to individual fatty acids but involve a coordinated shift in the overall lipid profile. In contrast, the absence of clear separation in the PCA of muscle supports the conclusion that IMF was less affected by seasonal fattening conditions. Thus, the multivariate analysis strengthens the idea that seasonal differences were robust in SAT and much weaker in IMF.

The neophytadiene results further support the hypothesis that differential levels of pasture intake are associated with the seasonal variation observed in this study. Neophytadiene has been described as a potential biomarker of grass consumption in Iberian pigs and related production systems, since it accumulates in adipose tissue when animals consume fresh pasture under extensive conditions [24,27]. Accordingly, the markedly higher neophytadiene concentrations found in winter-spring pigs compared with spring–summer and summer–autumn pigs are consistent with greater access to and intake of grass during the winter–spring period. Overall, the convergence of neophytadiene data with fatty acid contents, nutritional quality indices, and PCA results strongly supports a pasture-related explanation for the seasonal differences, particularly in SAT.

Some limitations of the present study should be considered when interpreting these findings. Nutritional quality indices only contribute a partial view of meat quality, as they are based on fatty acid composition and do not reflect other nutritional or sensory attributes. In addition, the seasonal differences observed should not be interpreted as the effect of a single independent variable, as season represents a composite factor that simultaneously encompasses multiple confounding variables, including pasture availability and quality, environmental temperature, and potential differences in animal activity. Therefore, the observed effects are likely the result of the combined influence of these interacting factors rather than any single component. Furthermore, pasture consumption was not directly measured at the individual level, so its role is inferred from tissue fatty acid and neophytadiene contents.

## 5. Conclusions

This study provides evidence that the seasonal fattening period may influence the lipid composition and nutritional quality of *cebo de campo* Iberian pigs reared under semi-extensive production conditions. Pigs fattened during the winter–spring period tended to show a more favorable overall lipid profile in nutritional terms compared to those fattened during the spring–summer and summer–autumn periods. The most pronounced differences were observed in subcutaneous adipose tissue rather than in intramuscular fat. Higher neophytadiene concentrations in winter–spring pigs further suggest a greater contribution of pasture intake during this period. These findings highlight the potential relevance of seasonal production conditions in shaping lipid quality in the *cebo de campo* system, suggesting that the timing of the fattening period could be considered a management factor to optimize product quality.

Further research should focus on accurately determining pasture availability during the finishing period and precisely quantifying individual intake, evaluating how these factors interact with environmental factors. As a result, their effects can be studied beyond meat composition and nutritional quality to include sensorial quality, as well as technological quality for the processing of dry-cured meat products.

## Figures and Tables

**Table 1:** Lipid nutritional indices and calculation formulas.

No.	Index	Full Name	Calculation Formula
1	n-6/n-3	Omega-6/Omega-3 Ratio	ΣPUFA n-6/ΣPUFA n-3
2	PUFA/SFA	Polyunsaturated/Saturated Fatty Acid Ratio	ΣPUFA/ΣSFA
3	MUFA/SFA	Monounsaturated/Saturated Fatty Acid Ratio	ΣMUFA/ΣSFA
4	UFA/SFA	Unsaturated/Saturated Fatty Acid Ratio	ΣUFA/ΣSFA
5	AI	Atherogenic Index	(C12:0 + 4 × C14:0 + C16:0)/(ΣMUFA + ΣPUFA n-6 + ΣPUFA n-3)
6	TI	Thrombogenic Index	(C14:0 + C16:0 + C18:0)/(0.5 × ΣMUFA + 0.5 × ΣPUFA n-6 + 3 × ΣPUFA n-3 + (n-3/n-6))
7	h/H	Hypocholesterolemic/Hypercholesterolemic Ratio	(C18:1 + ΣPUFA)/(C12:0 + C14:0 + C16:0)
8	HFI	Healthy Fatty Index	(2 × ΣMUFA + 4 × ΣPUFA n-6 + 8 × ΣPUFA n-3 + (n-3/n-6))/(ΣSFA + 0.5 × ΣMUFA + 0.25 × ΣPUFA n-6 + 0.125 × ΣPUFA n-3 + (n-3/n-6))

**Table 2:** Effect of the seasonal fattening period of Iberian pigs on the fatty acid content (mg/g) of subcutaneous adipose tissue and the *longissimus lumborum* muscle.

	Winter–Spring			Spring–Summer			Summer–Autumn			Effect
	Subcutaneous adipose tissue (SAT)									
Saturated										
C14:0	11.25	±	0.09 a	12.66	±	0.11 b	12.44	±	0.11 b	***
C16:0	197.58	±	0.91 a	212.60	±	1.00 b	214.45	±	0.80 b	***
C17:0	3.56	±	0.08	3.60	±	0.07	3.46	±	0.06	ns
C18:0	98.87	±	1.14 a	111.83	±	0.96 b	114.14	±	1.03 b	***
C20:0	2.05	±	0.03 a	2.04	±	0.02 a	2.23	±	0.04 b	***
Monounsaturated										
C16:1	22.67	±	0.32	22.04	±	0.25	21.95	±	0.24	ns
C17:1	4.02	±	0.09 b	3.80	±	0.07 ab	3.58	±	0.06 a	***
C18:1 n-9	453.07	±	1.62 b	430.99	±	1.44 a	427.40	±	1.32 a	***
C20:1 n-9	14.13	±	0.23 b	13.05	±	0.18 a	12.67	±	0.15 a	***
Polyunsaturated										
C20:2 n-9	5.82	±	0.10 b	5.05	±	0.07 a	4.89	±	0.06 a	***
C18:2 n-6	71.42	±	0.78 b	67.33	±	0.59 a	67.67	±	0.63 a	***
C20:3 n-6	0.86	±	0.02 b	0.79	±	0.01 a	0.78	±	0.01 a	*
C20:4 n-6	1.20	±	0.02 a	1.18	±	0.02 a	1.28	±	0.02 b	***
C18:3 n-3	5.43	±	0.11 c	4.22	±	0.05 a	4.44	±	0.05 b	***
C20:3 n-3	1.76	±	0.04 b	1.29	±	0.02 a	1.37	±	0.02 a	***
	*Longissimus lumborum* muscle (IMF)									
Saturated										
C14:0	0.87	±	0.05 b	0.60	±	0.02 a	0.64	±	0.02 a	***
C16:0	16.05	±	0.93 b	11.01	±	0.40 a	11.68	±	0.40 a	***
C17:0	0.10	±	0.01 b	0.08	±	0.00 a	0.08	±	0.00 a	***
C18:0	6.79	±	0.40 b	4.70	±	0.18 a	5.16	±	0.18 a	***
C20:0	0.13	±	0.01 b	0.09	±	0.00 a	0.09	±	0.00 a	***
Monounsaturated										
C16:1	2.77	±	0.16 b	2.02	±	0.07 a	2.08	±	0.07 a	***
C17:1	0.14	±	0.01 b	0.12	±	0.00 a	0.12	±	0.00 a	*
C18:1 n-9	30.51	±	1.62 b	20.93	±	0.72 a	21.72	±	0.69 a	***
C20:1 n-9	0.52	±	0.03 b	0.37	±	0.01 a	0.38	±	0.01 a	***
Polyunsaturated										
C20:2 n-9	0.09	±	0.01 b	0.07	±	0.00 a	0.07	±	0.00 a	**
C18:2 n-6	2.33	±	0.10 b	2.02	±	0.05 a	2.21	±	0.05 ab	*
C20:3 n-6	0.07	±	0.01	0.07	±	0.00	0.07	±	0.00	ns
C20:4 n-6	0.36	±	0.02	0.38	±	0.01	0.40	±	0.01	ns
C18:3 n-3	0.15	±	0.01 b	0.11	±	0.00 a	0.12	±	0.01 a	***
C20:3 n-3	0.06	±	0.00	0.05	±	0.00	0.05	±	0.00	ns

Values are presented as the means ± standard error. a, b, c: different letters within the same row indicate significant differences among groups (Tukey’s test, *p* < 0.05). Significance levels: ns, not significant (*p* > 0.05); *, *p* < 0.05; **, *p* < 0.01; ***, *p* < 0.001.

**Table 3:** Effect of the seasonal fattening period of Iberian pigs on the nutritional lipid quality indices of subcutaneous adipose tissue and intramuscular fat of m. *longissimus lumborum*.

	Winter–Spring			Spring–Summer			Summer–Autumn			Effect
Subcutaneous adipose tissue (SAT)										
Fat (mg/g)	893.69	±	1.83	892.47	±	1.50	892.74	±	1.43	ns
SFA (mg/g)	313.32	±	1.76 a	342.73	±	1.79 b	346.71	±	1.59 b	***
MUFA (mg/g)	493.89	±	1.78 b	469.87	±	1.61 a	465.60	±	1.44 a	***
PUFA (mg/g)	86.48	±	0.92 b	79.86	±	0.69 a	80.43	±	0.74 a	***
UFA (mg/g)	580.37	±	2.25 b	549.73	±	1.83 a	546.03	±	1.71 a	***
n-6 (mg/g)	73.48	±	0.80 b	69.30	±	0.60 a	69.73	±	0.65 a	***
n-3 (mg/g)	7.19	±	0.13 c	5.51	±	0.06 a	5.81	±	0.07 b	***
n-6/n-3	10.37	±	0.17 a	12.63	±	0.08 c	12.07	±	0.09 b	***
PUFA/SFA	0.28	±	0.00 b	0.23	±	0.00 a	0.23	±	0.00 a	***
MUFA/SFA	1.58	±	0.01 b	1.38	±	0.01 a	1.35	±	0.01 a	***
UFA/SFA	1.86	±	0.02 b	1.61	±	0.01 a	1.58	±	0.01 a	***
AI	0.42	±	0.00 a	0.48	±	0.00 b	0.48	±	0.00 b	***
TI	1.01	±	0.01 a	1.18	±	0.01 b	1.20	±	0.01 b	***
h/H	2.59	±	0.02 a	2.28	±	0.02 b	2.24	±	0.01 b	***
HFI	2.27	±	0.01 b	2.07	±	0.01 a	2.06	±	0.01 a	***
*Longissimus lumborum* muscle (IMF)										
Fat (mg/g)	60.93	±	3.28 b	42.60	±	1.44 a	44.88	±	1.41 a	***
SFA (mg/g)	23.94	±	1.39 b	16.48	±	0.60 a	17.66	±	0.61 a	***
MUFA (mg/g)	33.94	±	1.80 b	23.43	±	0.81 a	24.30	±	0.77 a	***
PUFA (mg/g)	3.06	±	0.13 b	2.69	±	0.06 a	2.92	±	0.05 ab	*
UFA (mg/g)	37.00	±	1.91 b	26.12	±	0.85 a	27.22	±	0.82 a	***
n-6 (mg/g)	2.76	±	0.11 b	2.46	±	0.05 a	2.68	±	0.06 ab	*
n-3 (mg/g)	0.21	±	0.01 b	0.16	±	0.01 a	0.17	±	0.01 a	***
n-6/n-3	14.60	±	0.53 a	17.12	±	0.40 b	16.92	±	0.42 b	***
PUFA/SFA	0.14	±	0.00 a	0.17	±	0.00 b	0.18	±	0.00 b	***
MUFA/SFA	1.44	±	0.02	1.43	±	0.02	1.39	±	0.03	ns
UFA/SFA	1.58	±	0.02	1.61	±	0.02	1.56	±	0.01	ns
AI	0.52	±	0.01	0.51	±	0.00	0.52	±	0.00	ns
TI	1.23	±	0.01	1.20	±	0.01	1.23	±	0.01	ns
h/H	2.02	±	0.03	2.06	±	0.02	2.03	±	0.02	ns
HFI	1.40	±	0.03 b	1.24	±	0.02 a	1.27	±	0.02 a	***

Values are presented as the means ± standard error. a, b, c: different letters within the same row indicate significant differences among groups (Tukey’s test, *p* < 0.05). Significance levels: ns, not significant (*p* > 0.05); *, *p* < 0.05; ***, *p* < 0.001. SFA: sum of all identified saturated fatty acids; MUFA: sum of all identified monounsaturated fatty acids; PUFA: sum of all identified polyunsaturated fatty acids; UFA: sum of all identified unsaturated fatty acids; n-6: sum of all identified n-6 fatty acids; n-3: sum of all identified n-3 fatty acids; n-6/n-3: ratio between total identified n-6 and n-3 fatty acids; AI: atherogenic index; TI: thrombogenic index; h/H: hypocholesterolemic/hypercholesterolemic ratio; HFI: healthy fatty index.

## Data Availability

The original contributions presented in this study are included in the article. Further inquiries can be directed to the corresponding author.

## Abbreviations

The following abbreviations are used in this manuscript:

AI	Atherogenic index
FAMEs	Fatty acid methyl esters
h/H	Hypocholesterolemic/hypercholesterolemic ratio
HFI	Healthy fatty index
IMF	Intramuscular fat
MUFAs	Monounsaturated fatty acids
NQS	National quality standard
PCA	Principal component analysis
PC1	First principal component
PC2	Second principal component
PUFAs	Polyunsaturated fatty acids
SAT	Subcutaneous adipose tissue
SFAs	Saturated fatty acids
TI	Thrombogenic index
UFAs	Unsaturated fatty acids
